# Editorial: Regulation of AMPA Receptors, From the Genetic to the Functional Level

**DOI:** 10.3389/fnsyn.2022.952564

**Published:** 2022-07-12

**Authors:** Alberto Ouro, Tak Pan Wong, Laura Jiménez-Sánchez

**Affiliations:** ^1^NeuroAging Group (NEURAL), Clinical Neurosciences Research Laboratories (LINCs), Health Research Institute of Santiago de Compostela (IDIS), Santiago de Compostela, Spain; ^2^Basic Neuroscience Division, Douglas Hospital Research Centre, Montreal, QC, Canada; ^3^Department of Psychiatry, McGill University, Montreal, QC, Canada; ^4^Instituto de Investigación Biosanitaria Ibs.GRANADA, Granada, Spain

**Keywords:** AMPAR, synaptic plasticity, neurodegeneration, neural remodeling, receptor tracking

AMPA receptors (AMPARs) are widely distributed in the central and peripheral nervous systems. They are mainly located in postsynaptic cells, but can also be found in pre-synaptic sites and glia processes. Upon glutamate stimulation, the postsynaptic AMPARs mediate the fast excitatory synaptic transmission which can be changed in an activity dependent manner and might involve a minority of Ca^2+^ permeable AMPARs. This Research Topic describes several new mechanisms that regulate AMPAR trafficking and plasticity; in addition to give novel insight to the function of AMPAR in the hypothalamus.

AMPARs are also major players in synaptic plasticity, in fact, long-term depression (LTD) and long-term potentiation (LTP) are determined by the number of postsynaptic receptors. These processes, which are altered in some neurodegenerative diseases, require not only the mobilization of AMPARs at the synaptic contacts but also the contribution of different glutamatergic receptors (Sanderson et al., 2011). It is well known that LTD can be induced by the activation of group I metabotropic glutamate receptors (mGluRs) in area CA1 of the hippocampus with glycine antagonist 3,5-dihydroxyphenylglycine (DHPG). This plasticity is necessary for normal brain functions, but the exact mechanism is unknown. In this Research Topic, Sanderson et al. have demonstrated two different mechanisms by which mGluRs induce LTD. *In vitro* studies at different concentrations of DHPG have shown that mGluRs can stimulate LTD through a presynaptically NMDA-dependent or an NMDA-independent mechanism involving AMPARs. These results are relevant to understanding synaptic regulatory mechanisms. AMPARs are tetrameric receptors whose surface expression is tightly regulated by neuronal activity. Together with the advance in imaging and molecular techniques for labeling AMPARs, recent findings support the contribution of AMPARs in extrasynaptic and subsynaptic domains to activity-dependent trafficking. In addition, sep-tagged AMPARs have allowed the imaging of these receptors during activity-dependent plasticity *in vivo*. In this Research Topic, Chater and Goda have provided a timely review on these topics.

Regulation of AMPAR subunits phosphorylation is one of the mechanisms used by the cells to control the function and the location of the receptor. Different signals are responsible for the phosphorylation of AMPAR and alterations in this phosphorylation status have been associated with different brain diseases, like Alzheimer's disease. One of the earliest manifestations of this disease, is a reduction of AMPARs in the synapse and altered synaptic plasticity. By imaging Phosphatase and Tensin homolog (PTEN), a phosphatase that is related to LTD, Gonzalez et al. have found that Alzheimer's disease patients showed a progressive increase in PTEN expression in the dentate gyrus, that is associated with lower synaptic density and AMPAR expression in synapses. These findings suggest an increase in PTEN could underlie synaptic failure and loss in Alzheimer's disease.

In the same line, it is well established that the phosphorylation state of intracellular domain of AMPAR is involved in synaptic plasticity, producing LTP or LTD. In fact, an increase in cell-surface insertion, in channel open probability and conductance, and induction of LTP have been detected after phosphorylation of GluA1 at S845 or S831. Proline-rich transmembrane protein 1 (PRRT1), also known as SynDIG4, is a component of native AMPAR complexes detected in multiple brain regions. Previous works described that deletion of PRRT1 leads to altered surface expression and phosphorylation status of AMPARs, in addition to impaired synaptic plasticity. In this Research Topic, Martin et al. demonstrated that PRRT1 regulates the phosphorylated state of AMPARs by direct interaction with AMPAR subunits, and with serine/threonine-protein phosphatase 2B (PP2B) during synaptic plasticity. These results identify the action model of PRRT1 to modulate synaptic plasticity.

Glutamate receptors are densely distributed in the central nervous systems. The different subunits of AMPARs have been found in the cortex, basal ganglia, olfactory regions, lateral septum or amygdala among others. However, most of the functional studies are focused only on the hippocampus, and its function in other brain areas has not been studied in such depth. Royo et al. have done extensive work on the function of AMPARs in the hypothalamus, and their implication in neuroendocrine function, body homeostasis, and social behavior.

The current Research Topic deepens the knowledge of the biology of AMPARs in neurotransmission and synaptic plasticity, which undoubtedly provides insights into the mechanisms associated with the dysfunction of these receptors in neurological disorders.

## Author Contributions

All authors listed have made a substantial, direct, and intellectual contribution to the work and approved it for publication.

## Conflict of Interest

The authors declare that the research was conducted in the absence of any commercial or financial relationships that could be construed as a potential conflict of interest.

## Publisher's Note

All claims expressed in this article are solely those of the authors and do not necessarily represent those of their affiliated organizations, or those of the publisher, the editors and the reviewers. Any product that may be evaluated in this article, or claim that may be made by its manufacturer, is not guaranteed or endorsed by the publisher.

## References

[B1] Sanderson T. M. Collingridge G. L. Fitzjohn S. M. (2011). Differential trafficking of AMPA receptors following activation of NMDA receptors and mGluRs. Mol. Brain. 4, 1–10. 10.1186/1756-6606-4-30/FIGURES/5 21794146 PMC3160366

